# Longitudinal Study of Diabetes and Depressive Symptoms Among Middle‐Aged and Older Adults in China: The Mediating Roles of Functional Limitations and Social Interaction

**DOI:** 10.1002/edm2.70378

**Published:** 2026-11-10

**Authors:** Yan Tao, Claire MacGilchrist, Ellen Kirwan, Joanne E. Hurst, Caroline McIntosh

**Affiliations:** ^1^ Discipline of Podiatric Medicine, School of Health Sciences University of Galway Ireland; ^2^ Alliance for Research and Innovation in Wounds, College of Medicine, Nursing & Health Sciences University of Galway Ireland

**Keywords:** depression, diabetes mellitus, functional status

## Abstract

**Introduction:**

Diabetes and depressive symptoms frequently coexist, but the pathways underlying their longitudinal associations remain unclear. This study examined their reciprocal associations and indirect pathways through functional limitations and social interaction.

**Methods:**

We analysed 6606 adults aged ≥ 45 years from the China Health and Retirement Longitudinal Study in 2013 (T1), 2015 (T2), and 2018 (T3). Diabetes was identified through self‐reported diagnosis or treatment; depressive symptoms were measured using the 10‐item Center for Epidemiologic Studies Depression Scale. Functional limitations and social interaction were assessed using activities of daily living scales and social activity frequency. Cross‐lagged panel models examined reciprocal associations and indirect pathways, with multigroup analyses evaluating invariance across gender and age. Logistic regression sensitivity analyses examined associations between depressive symptoms and incident diabetes.

**Results:**

In the full reciprocal model, direct associations between diabetes and depressive symptoms were significant during T1–T2 in both directions, but neither was significant during T2–T3. Logistic sensitivity analyses supported the depressive symptoms‐to‐diabetes association only during T1–T2, whereas both reverse adjacent‐wave paths were significant in the model without mediators. The direct T1–T3 association was significant only from diabetes to depressive symptoms. Statistically significant indirect associations from T1 diabetes to T3 depressive symptoms were observed through T2 functional limitations and reduced social interaction. Reverse indirect effects were statistically significant but very small. The structural pathways were broadly invariant across gender and age groups.

**Conclusion:**

The findings suggest reciprocal prospective associations between diabetes and depressive symptoms, but evidence for depressive symptoms predicting diabetes varied across models and follow‐up intervals.

## Graphical abstract

Among 6606 middle‐aged and older Chinese adults, diabetes and depressive symptoms showed reciprocal prospective associations that varied across follow‐up intervals. Functional limitations and reduced social interaction statistically supported indirect pathways from T1 diabetes to T3 depressive symptoms, whereas reverse indirect effects were negligible.

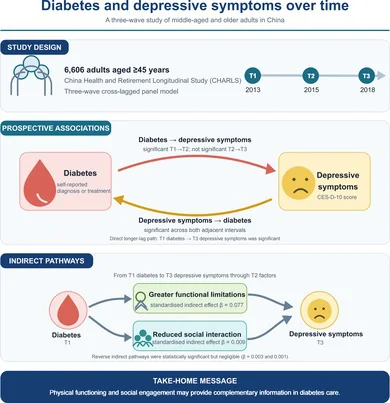

## 1 Introduction

Diabetes and depression are two of the most prevalent chronic conditions worldwide, and their coexistence has become an important public health concern [1, 2]. A growing body of evidence suggests a bidirectional link between diabetes and depression, in which each condition increases the risk of developing the other [2, 3, 4]. Among individuals with Type 2 diabetes, the risk of developing depression is 15%–24% higher compared to those without diabetes [3, 5]. When considering both Type 1 and Type 2 diabetes, individuals with diabetes have twice the odds of experiencing depression compared to individuals without diabetes [1]. Conversely, individuals with depression face a higher risk of developing diabetes, with the pooled relative risk ranging from 1.32 for Type 2 diabetes to 1.41–1.60 for diabetes overall [3, 6].

Most existing research has focused on estimating these associations, but the pathways between diabetes and depression remain insufficiently understood. To explain these complex interconnections, Katon [7] proposed a biopsychosocial conceptual model linking biobehavioural factors, physiological dysfunction, reduced quality of life, functional impairment and healthcare access to the onset and progression of both chronic diseases and depression. This model emphasises that the interconnection of diabetes and depressive symptoms is not driven by a single pathway, but rather by interrelated behavioural and functional processes [7]. Within this framework, functional limitations and social interaction are particularly important. Functional limitations reflect the physical consequences of chronic disease, whereas social interaction represents a key behavioural and psychosocial dimension influencing both mental health and disease management. Guided by this model, the present study aims to explore the bidirectional association between diabetes and depressive symptoms and investigate the indirect pathways through functional limitations and social interaction.

Functional limitations are common in people with diabetes and typically present as difficulties in performing basic and instrumental activities of daily living, with important consequences for physical health and daily functioning [8, 9]. According to recent global estimates, diabetes is now the third leading contributor to disability worldwide [10]. In China, 12.4% of individuals with diabetes reported functional impairments, increasing with age [11]. In a U.S. cohort, individuals with diabetes were found to have a two‐ to three‐fold higher likelihood of experiencing limitations in basic physical activities, including walking a quarter mile, climbing stairs, and household tasks [12]. Those with comorbid mental disorders (e.g., depression, anxiety) showed greater functional decline than those with diabetes alone [12]. A British population‐based cross‐sectional study indicated that, after adjusting for functional limitations, the association between diabetes and depressive symptoms became non‐significant [9], suggesting that functional limitations may act as a key mechanism linking the two conditions.

Reduced social interaction is another potential pathway linking diabetes and depressive symptoms. In the present study, social interaction refers specifically to participation in social activities, reflecting the behavioural dimension of social engagement and an important indicator of individual social functioning. Individuals with diabetes have been shown to participate less frequently in social activities and experience greater social disconnection than those without diabetes. For example, a 13.5‐year prospective study in the UK reported lower levels of social participation among people with diabetes [13]. Reduced social participation has consistently been associated with an increased risk of depressive symptoms [14, 15, 16]. Over 4 years of follow‐up, older adults who were less involved in social activities showed a 45% greater likelihood of subsequent depressive symptoms [16]. Conversely, an ecological momentary assessment study reported that individuals with more severe depressive symptoms engaged in fewer leisure activities, participated less in work, and were more likely to remain inactive [17], suggesting that depression may further reduce social participation. Emerging evidence suggests that limited social interaction is associated with an increased risk of diabetes [13, 15, 18]. Mendelian randomisation analyses from large European and Asian cohorts further indicate that greater social engagement with friends or family may reduce the risk of Type 2 diabetes [13]. Beyond its role in diabetes onset, social interaction has also been associated with diabetes‐related complications, including acute kidney injury [19] and cardiovascular disease [20]. These findings are also consistent with Katon's biopsychosocial model [7], in which social interaction represents a modifiable behavioural process linking physical and psychological health.

Functional limitations and social interaction are closely interconnected and may jointly contribute to the reciprocal relationship between diabetes and depressive symptoms. Functional limitations may reduce opportunities for social interaction by restricting mobility, independence, and engagement in community activities. For instance, a study by Liu et al. [21] showed that older Chinese adults with greater functional limitations participated less frequently in social activities. In turn, reduced social interaction has been shown to accelerate functional decline in a cohort study conducted in Japan [22], thereby creating a reinforcing cycle that may contribute to both diabetes progression and worsening depressive symptoms. Although previous studies have examined functional limitations or social participation separately, few have simultaneously investigated their mediating roles within a unified model while accounting for the reciprocal relationship between diabetes and depressive symptoms. To our knowledge, no previous longitudinal study has evaluated these two mediators simultaneously using a bidirectional analytical framework.

The primary objective of this study was to examine the longitudinal bidirectional association between diabetes and depressive symptoms. The secondary objective was to investigate indirect pathways through functional limitations and social interaction in a nationally representative sample from the China Health and Retirement Longitudinal Study (CHARLS).

## 2 Methods

### 2.1 Data Sources and Sample

This study used data from CHARLS, a nationally representative cohort designed to collect data on Chinese residents aged ≥ 45 years to support analyses of population ageing issues in China [23]. The baseline CHARLS survey (Wave 1) was conducted in 2011 using a nationally representative sample drawn from 150 county‐level units and 450 village‐level communities across China. Follow‐up assessments were completed every two to 3 years using self‐report questionnaires [23]. CHARLS includes rich data on demographics, physical and mental health status, retirement, and medical service utilisation. The CHARLS study was approved by the Institutional Review Board of Peking University (Approval No. IRB00001052‐11015) and was conducted in accordance with relevant ethical standards and the principles of the Declaration of Helsinki. Written informed consent was obtained from all participants before participation [23]. This study was reported in accordance with the Strengthening the Reporting of Observational Studies in Epidemiology (STROBE) guidelines.

In the present study, data from Wave 2 (2013, T1), Wave 3 (2015, T2), and Wave 4 (2018, T3) surveys were used. Participants were eligible for inclusion if they participated in all three waves and had complete data on the key variables, including diabetes, functional limitations, social interaction, and depressive symptoms. Of the 19,695 individuals who completed the Wave 2 survey, 4312 were excluded because they did not participate in all three waves, and a further 8777 were excluded due to missing data on key variables. Our final analytical sample included 6606 participants. The detailed inclusion and exclusion process is presented in Figure S1.

Compared with participants who were excluded from the analysis (*n* = 13,089), those retained in the final analytical sample were younger and reported fewer depressive symptoms at baseline. They were also more likely to be male, married or cohabiting, reside in urban areas, and have higher educational attainment. In addition, retained participants had a higher mean body mass index (BMI), were more likely to smoke and consume alcohol, and had a lower prevalence of diabetes than excluded participants (all *p* < 0.001).

### 2.2 Measures

#### 2.2.1 Sociodemographic Variables

All sociodemographic characteristics were collected at T1 and included age (45–59, ≥ 60), gender (female, male), marital status (no spouse/living alone, cohabitation/married), residence (urban, rural), education (illiterate, primary school, middle school, high school and above), BMI (< 18.5, 18.5–23.9, 24.0–27.9, ≥ 28.0), current smoking (no, yes), and current alcohol use (no, yes).

### 2.3 Diabetes

Diabetes was identified by self‐reported physician diagnosis and/or current use of diabetes medication, based on the questions: ‘Have you been diagnosed with diabetes by a doctor?’ and ‘Are you now taking any of the following treatments to treat or control your diabetes?’

#### 2.3.1 Depressive Symptoms

Depressive symptoms were measured using the 10‐item Center for Epidemiologic Studies Depression Scale (CES‐D10) [24], a widely used screening instrument for depressive symptoms. The scale comprises 10 items assessing depressive mood and affect over the previous week. Responses to each item range from 0 (‘rarely or none of the time, < 1 day’) to 3 (‘most or all of the time, 5–7 days’), with the two positively worded items (items 5 and 8) reverse‐scored. Total scores ranged from 0 to 30, where higher scores indicate more severe depressive symptoms. In line with previous studies [25], a CES‐D10 score ≥ 10 was used to indicate probable depressive caseness. The CES‐D10 demonstrated good internal consistency at baseline in the present study (Cronbach's *α* = 0.83).

#### 2.3.2 Functional Limitations

Functional limitations were evaluated using the combined Basic Activities of Daily Living (BADL) and Instrumental Activities of Daily Living (IADL) scales [26], as described in previous studies [27]. The BADL component comprised six activities (dressing, bathing, eating, getting in and out of bed, toileting, and bladder and bowel control), whereas the IADL component included five activities (household chores, cooking, shopping, financial management, and medication management). Responses to each of the 11 items were scored on a four‐point scale ranging from 1 (‘No, I don't have any difficulty’) to 4 (‘I cannot do it’). Scores from all items were summed to produce a total score ranging from 11 to 44, with higher values indicating poorer physical functioning and greater functional limitations. The combined BADL/IADL measure demonstrated acceptable internal consistency at baseline (Cronbach's *α* = 0.78).

#### 2.3.3 Social Interaction

Social interaction was quantified according to frequency of engagement in 10 activities over the past month based on previous studies [27]. These activities included meeting friends, playing Ma‐Jong, helping others, exercising, participating in community activities, volunteering or charity work, caring for sick individuals, attending educational or training programmes, investing in stocks, and using the internet. Each activity was scored on a four‐point scale ranging from 0 (“none”) to 3 (“almost daily”). Scores across all activities were summed to generate an overall score ranging from 0 to 30, with higher scores indicating greater social interaction. The scale demonstrated acceptable internal consistency at baseline in the present study (Cronbach's α = 0.73).

### 2.4 Statistical Analysis

Normally distributed continuous variables were summarised as means and standard deviations (SD), whereas categorical variables were described as frequencies and percentages. Baseline group comparisons and Spearman correlation analyses were conducted using SPSS v30.0. To assess potential common method bias resulting from self‐reported data, Harman's single‐factor test was performed at each wave, with more than 40% of the variance explained by the first factor indicating possible bias [28].

A three‐wave cross‐lagged panel model (CLPM) was conducted using Mplus v8.3 to test the longitudinal bidirectional relationship between diabetes and depressive symptoms, and the indirect pathways through functional limitations and social interaction [29]. Model 0 evaluated the bidirectional cross‐lagged paths between diabetes and depressive symptoms. Model 1 (stability model) included only autoregressive paths and within‐time correlations, serving as the baseline model for evaluating subsequent mediation models. Models 2 and 3 separately examined the diabetes‐to‐depressive symptoms and depressive symptoms‐to‐diabetes pathways through functional limitations and social interaction. Finally, Model 4 (full reciprocal model) included all reciprocal direct and indirect pathways. All models were adjusted for age, gender, marital status, BMI, current smoking, current alcohol use, education level, and residence. Missing BMI values were replaced with the sample mean before modelling. Age and BMI were entered as continuous covariates. Diabetes was coded as a binary observed variable (0 = no diabetes; 1 = diabetes) and was not declared as a categorical outcome. The models were estimated using maximum likelihood (ML). Indirect effects were assessed using a bias‐corrected bootstrap procedure with 5000 resamples and were considered statistically supported when the corresponding 95% confidence interval (CI) did not include zero. All path coefficients are reported as standardised estimates.

Model fit was evaluated using standard indices, including the Comparative Fit Index (CFI > 0.90), Tucker–Lewis Index (TLI > 0.90), Root Mean Square Error of Approximation (RMSEA < 0.08), and Standardised Root Mean Square Residual (SRMR < 0.08) [30]. Given the sensitivity of chi‐square (*χ* ^2^) statistics to large sample sizes, the *χ* ^2^ values and degrees of freedom were reported for reference only. Nested models were compared using *χ* ^2^ difference tests, interpreted in conjunction with other fit indices.

Two sets of sensitivity analyses were conducted. First, sensitivity analyses of the depressive symptoms‐to‐diabetes direction were conducted using multivariable logistic regression, thereby modelling diabetes explicitly as a binary outcome. For the T1–T2 interval, participants with diabetes at T1 were excluded, and diabetes at T2 was modelled as the binary outcome. For the T2–T3 interval, participants with diabetes at T1 or T2 were excluded, and diabetes at T3 was modelled as the binary outcome. Depressive symptoms, functional limitations, and social interaction measured at the beginning of each interval were entered as predictors with adjustment for age, gender, marital status, BMI, current smoking, current alcohol use, education level, and residence. Adjusted odds ratios (ORs) and 95% confidence intervals were reported. These analyses assessed interval‐specific prospective associations with incident diabetes. Second, to assess the robustness of the diabetes‐to‐depressive symptoms pathways, Model 2 was re‐estimated after excluding participants with probable depression at baseline.

We also conducted multigroup analyses to examine model invariance by gender and age (< 60 vs. ≥ 60 years). The age threshold followed the National Bureau of Statistics of China definition of older adults [31]. For each grouping variable, a constrained model in which the structural paths were set equal across groups was compared with an unconstrained model in which these paths were freely estimated. Evidence of invariance was considered supported when changes in the primary approximate fit indices did not exceed the thresholds of ΔCFI ≤ 0.010, ΔRMSEA ≤ 0.015, and ΔSRMR ≤ 0.010 [32]. Changes in TLI were reported as supplementary fit information but were not used as a primary decision criterion [32]. All statistical tests were two‐sided, and *p* < 0.05 was considered statistically significant.

## 3 Results

### 3.1 Preliminary Analysis

Harman's single‐factor test indicated no evidence of common method bias, with variance explained by the first factor at 13.5%, 11.5%, and 23.8% across the three waves, all below the 40% threshold.

Baseline characteristics of participants are presented in Table 1. Among the 6606 participants, the mean age was 57.6 (SD = 8.2) years, 60.6% were aged 45–59 years, and 45.6% were female. Compared with participants without diabetes, those with diabetes were older, more likely to be female, had lower education levels, and reported more severe depressive symptoms (Table 1). The number of participants with diabetes increased from 357 (5.4%) at T1 to 528 (8.0%) at T2 and 851 (12.9%) at T3. Probable depression caseness was identified in 27.2% (1799/6606) of the total sample and 40.6% (145/357) of participants with diabetes at T1. These corresponding proportions were 28.5% (1884/6606) and 42.0% (222/528) at T2, and 31.9% (2110/6606) and 40.4% (344/851) at T3, respectively.

**TABLE 1:** Baseline characteristics of participants.

Variables	Overall (*N* = 6606)	No diabetes (*n* = 6249)	Diabetes case (*n* = 357)	*p* value^a^
Depressive symptoms score	7.19 ± 5.41	7.09 ± 5.37	9.02 ± 5.84	**< 0.001**
Depression case	1799 (27.2)	1654 (26.5)	145 (40.6)	**< 0.001**
Age (years)	57.61 ± 8.23	57.43 ± 8.21	60.77 ± 8.12	**< 0.001**
Age group (years)				**< 0.001**
45–59	4002 (60.6)	3843 (61.5)	159 (44.5)	
≥ 60	2604 (39.4)	2406 (38.5)	198 (55.5)	
Gender				**0.003**
Female	3012 (45.6)	2822 (45.2)	190 (53.2)	
Male	3594 (54.4)	3427 (54.8)	167 (46.8)	
Marital status				0.072
No spouse or living alone	520 (7.9)	483 (7.7)	37 (10.4)	
Cohabitation or married	6086 (92.1)	5766 (92.3)	320 (89.6)	
Residence				0.454
Rural	2761 (41.8)	2605 (41.7)	156 (43.7)	
Urban	3845 (58.2)	3644 (58.3)	201 (56.3)	
Education level				**0.034**
Illiterate	1623 (24.6)	1515 (24.2)	108 (30.3)	
Primary school	1757 (26.6)	1662 (26.6)	95 (26.6)	
Middle school	2038 (30.9)	1934 (30.9)	104 (29.1)	
High school and above	1188 (18.0)	1138 (18.2)	50 (14.0)	
BMI (kg/m^2^)	24.29 ± 3.71	24.29 ± 3.71	24.36 ± 3.68	0.744
BMI group (kg/m^2^)				0.945
< 18.5	231 (3.5)	217 (3.5)	14 (3.9)	
18.5–23.9	2916 (44.1)	2761 (44.2)	155 (43.4)	
24–27.9	2244 (34.0)	2119 (33.9)	125 (35.0)	
≥ 28	945 (14.3)	895 (14.3)	50 (14.0)	
Missing	270 (4.1)	257 (4.1)	13 (3.6)	
Current alcohol use				0.414
Yes	3266 (49.4)	3097 (49.6)	169 (47.3)	
No	3340 (50.6)	3152 (50.4)	188 (52.7)	
Current smoking				0.113
Yes	3119 (47.2)	2965 (47.4)	154 (43.1)	
No	3487 (52.8)	3284 (52.6)	203 (56.9)	

*Note:* Data are presented as mean ± SD or *n* (%). Bold *p* values indicate statistically significant differences between participants with and without diabetes (*p* < 0.05).

^a^ Two‐sided *p* values were obtained using a two‐sample *t*‐test for continuous data and the *χ* ^2^ test for categorical data.

Table 2 presents the bivariate correlations among diabetes status, functional limitations, social interaction, and depressive symptoms across three time points. Diabetes was positively associated with functional limitations and depressive symptoms, and social interaction was negatively correlated with all three variables across waves. Functional limitations and depressive symptoms also showed a positive association.

**TABLE 2:** Bivariate correlations between the main variables across the three waves (*N* = 6606).

Variables	1	2	3	4	5	6	7	8	9	10	11	12
1. T1 Diabetes	1											
2. T2 Diabetes	0.81**	1										
3. T3 Diabetes	0.62**	0.77**	1									
4. T1 Functional limitations	0.09**	0.11**	0.11**	1								
5. T2 Functional limitations	0.10**	0.11**	0.12**	0.50**	1							
6. T3 Functional limitations	0.11**	0.12**	0.12**	0.44**	0.51**	1						
7. T1 Social interaction	−0.03*	−0.04**	−0.03**	−0.07**	−0.08**	−0.08**	1					
8. T2 Social interaction	−0.05**	−0.06**	−0.07**	−0.08**	−0.07**	−0.09**	0.37**	1				
9. T3 Social interaction	−0.05**	−0.05**	−0.04**	−0.08**	−0.09**	−0.11**	0.33**	0.37**	1			
10. T1 Depressive symptoms	0.08**	0.09**	0.10**	0.27**	0.25**	0.22**	−0.09**	−0.09**	−0.11**	1		
11. T2 Depressive symptoms	0.08**	0.10**	0.11**	0.22**	0.33**	0.25**	−0.08**	−0.09**	−0.12**	0.55**	1	
12. T3 Depressive symptoms	0.11**	0.12**	0.11**	0.22**	0.25**	0.32**	−0.09**	−0.10**	−0.11**	0.50**	0.53**	1

* *p* < 0.05.

** *p* < 0.01.

### 3.2 Cross‐Lagged Panel Models and Mediation Analysis

To test the bidirectional relationship between diabetes and depressive symptoms, Model 0 was estimated and showed excellent fit (CFI = 0.996, TLI = 0.998, RMSEA = 0.017, SRMR = 0.003). As shown in Figure 1, the adjacent‐wave paths indicated that T1 diabetes significantly predicted T2 depressive symptoms (*β* = 0.537, *p* < 0.001), whereas T2 diabetes did not significantly predict T3 depressive symptoms (*β* = 0.444, *p* = 0.299). In the reverse direction, T1 depressive symptoms significantly predicted T2 diabetes (*β* = 0.026, *p* < 0.001), and T2 depressive symptoms significantly predicted T3 diabetes (*β* = 0.019, *p* = 0.043). Regarding the direct longer‐lag paths, T1 diabetes was significantly associated with T3 depressive symptoms (*β* = 0.443, *p* = 0.024), whereas the direct path from T1 depressive symptoms to T3 diabetes was not statistically significant (*β* = 0.014, *p* = 0.151).

**FIGURE 1:**
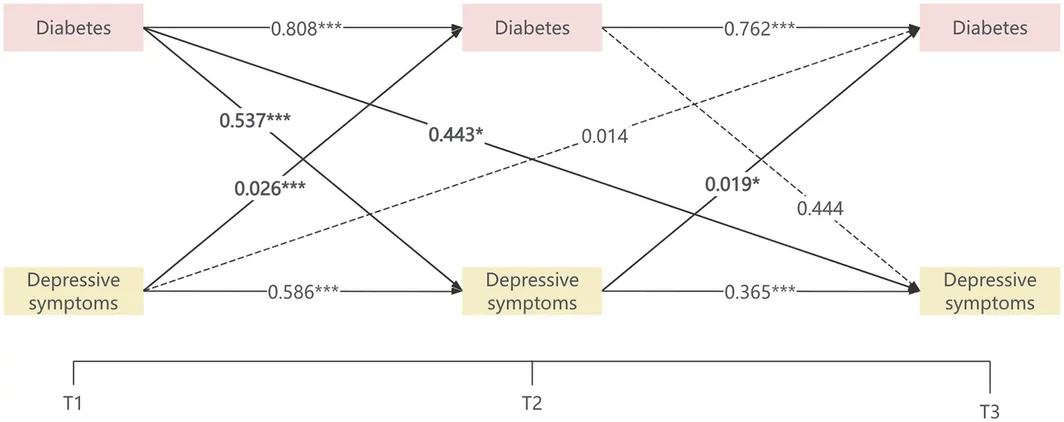
Cross‐lagged panel model for diabetes and depressive symptoms (Model 0). Single‐headed arrows represented regression paths. Double‐headed arrows represented correlations. The solid line represents a significant path, and the dashed line represents a non‐significant path. All path coefficients were standardised. *p < 0.05, **p < 0.01, ***p < 0.001.

Then, a nested comparison approach was applied to evaluate model fit and identify the best‐fitting model after including the mediators. The model fit indices are presented in Table 3. All models achieved acceptable fit according to the predefined criteria (CFI and TLI > 0.90; RMSEA and SRMR < 0.08). In the stability model (Model 1), all autoregressive paths were statistically significant (*p* < 0.001), with standardised estimates ranging from 0.320 to 0.810. Model 2, which incorporated the diabetes‐to‐depressive symptoms mediation pathways, and Model 3, which incorporated the depressive symptoms‐to‐diabetes mediation pathways, each showed improved model fit relative to Model 1. The full reciprocal model (Model 4), which simultaneously incorporated both sets of mediation pathways, showed further improvements in model fit relative to Models 2 and 3 and was therefore retained as the final model. The standardised path coefficients for Models 1–3 are provided in Figures S2–S4.

**TABLE 3:** Model fit indices and model comparisons.

Model	Model fit indices					Model comparisons						
	*χ* ^2^ (df)	RMSEA	CFI	TLI	SRMR	Comparison	Δ*χ* ^2^ (Δdf)	*p*	ΔRMSEA	ΔCFI	ΔTLI	ΔSRMR
Model 1	848.321 (38)	0.057	0.969	0.924	0.040							
Model 2	310.740 (23)	0.044	0.989	0.956	0.022	Model 2 versus 1	537.581 (15)	< 0.001	0.013	0.020	0.032	0.018
Model 3	217.637 (23)	0.036	0.992	0.970	0.020	Model 3 versus 1	630.684 (15)	< 0.001	0.021	0.023	0.046	0.020
Model 4	44.529 (10)	0.023	0.999	0.988	0.005	Model 4 versus 2	266.211 (13)	< 0.001	0.021	0.010	0.032	0.017
						Model 4 versus 3	173.108 (13)	< 0.001	0.013	0.007	0.018	0.015

*Note:* Model 1: stability model; Model 2: Mediation model (from diabetes → depressive symptoms); Model 3: Mediation model (from depressive symptoms → diabetes); Model 4: Full mediation model.

Abbreviations: *χ* ^2^, chi‐square; Δ, change in parameter; CFI, comparative fit index; df, degrees of freedom; RMSEA, root mean square error of approximation; SRMR, standardised root mean square residual; TLI, tucker–lewis index.

In the final model (Model 4; Figure 2), T1 diabetes significantly predicted greater functional limitations (*β* = 0.383, *p* = 0.007) and lower social interaction (*β* = −0.227, *p* = 0.001) at T2, both of which subsequently predicted more severe depressive symptoms at T3 (functional limitations: *β* = 0.201, *p* < 0.001; social interaction: *β* = −0.038, *p* < 0.001). Similarly, T1 depressive symptoms significantly predicted greater functional limitations (*β* = 0.113, *p* < 0.001) and lower social interaction (*β* = −0.044, *p* < 0.001) at T2, which subsequently predicted diabetes at T3 (functional limitations: *β* = 0.030, *p* = 0.004; social interaction: *β* = −0.018, *p* = 0.002). Functional limitations and social interaction also exhibited significant reciprocal associations across the two adjacent intervals (*β* range: −0.043 to −0.020; all *p* < 0.05), indicating that greater social interaction was associated with fewer functional limitations, and vice versa.

**FIGURE 2:**
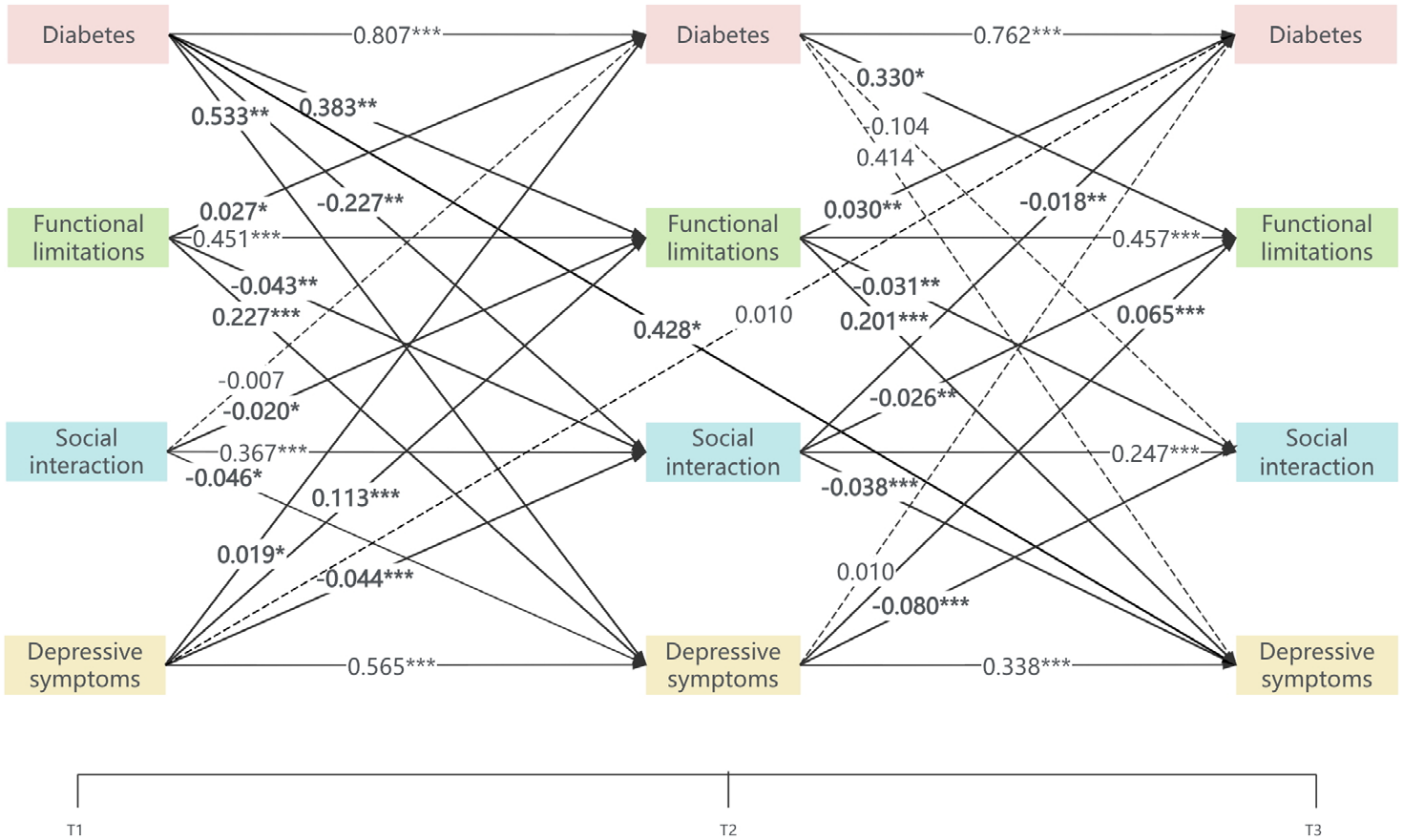
Cross‐lagged panel model for diabetes, functional limitations, social interaction and depressive symptoms (Model 4). Single‐headed arrows represented regression paths. Double‐headed arrows represented correlations. The solid line represents a significant path, and the dashed line represents a non‐significant path. All path coefficients were standardised. *p < 0.05, **p < 0.01, ***p < 0.001.

Bootstrap analysis supported significant indirect effects of T1 diabetes on T3 depressive symptoms through T2 functional limitations (standardised: β = 0.077, 95% CI [0.017, 0.138], *p* = 0.012) and T2 social interaction (standardised: β = 0.009, 95% CI [0.003, 0.025], *p* = 0.014). Very small but statistically significant indirect effects of T1 depressive symptoms on T3 diabetes were also observed through T2 functional limitations (standardised: β = 0.003, 95% CI [0.001, 0.006]; unstandardised: B = 2.12 × 10^−4^, 95% CI [7.40 × 10^−5^, 4.07 × 10^−4^], *p* = 0.012) and social interaction (standardised: β = 0.001, 95% CI [0.000, 0.002]; unstandardised: B = 4.90 × 10^−5^, 95% CI [1.60 × 10^−5^, 1.02 × 10^−4^], *p* = 0.022). Because the lower bounds of the standardised 95% CI for these reverse indirect effects were affected by rounding, the corresponding unstandardised estimates are additionally reported with greater precision.

### 3.3 Sensitivity and Multigroup Analyses

In the interval‐specific logistic regression analyses, higher T1 depressive symptom scores were associated with increased odds of incident diabetes at T2 (adjusted OR = 1.032, 95% CI [1.004, 1.060], *p* = 0.022), whereas T2 depressive symptoms were not significantly associated with incident diabetes at T3 (adjusted OR = 1.011, 95% CI [0.989, 1.034], *p* = 0.311). For the depressive symptoms‐to‐diabetes association, statistical support was observed only during T1–T2 in the logistic analyses and for the corresponding direct paths in Model 4, whereas Model 0 showed significant paths across both adjacent‐wave intervals. Greater functional limitations predicted incident diabetes during both intervals (T1–T2: adjusted OR = 1.103, 95% CI [1.042, 1.168], *p* = 0.001; T2–T3: adjusted OR = 1.083, 95% CI [1.036, 1.132], *p* < 0.001), while greater social interaction was associated with lower odds of incident diabetes only during T2–T3 (adjusted OR = 0.950, 95% CI [0.915, 0.986], *p* = 0.007).

In the diabetes‐to‐depressive symptoms direction, re‐estimation of Model 2 after excluding participants with probable depression at T1 yielded an acceptable model fit, with the direction and statistical significance of the principal cross‐lagged and indirect pathways largely unchanged, supporting the robustness of the primary findings (Table S1 and Figure S5).

Multigroup analyses showed that both the unconstrained and constrained models showed good fit across gender and age groups (Tables S2 and S3). Changes in the approximate fit indices were below the prespecified invariance thresholds for gender (ΔCFI = 0.003, ΔRMSEA = 0.008, and ΔSRMR = 0.003) and age (ΔCFI = 0.002, ΔRMSEA = 0.005, and ΔSRMR = 0.002). These findings supported the invariance of the structural pathways across gender and age subgroups.

## 4 Discussion

This study examined the longitudinal reciprocal associations between diabetes and depressive symptoms and the potential indirect pathways involving functional limitations and social interaction among middle‐aged and older Chinese adults. Prospective associations between diabetes and depressive symptoms were observed, but statistical support for the depressive symptoms‐to‐diabetes direction was less consistent across models and follow‐up intervals. Indirect associations from T1 diabetes to T3 depressive symptoms through functional limitations and social interaction were observed, whereas the corresponding reverse indirect effects were statistically significant but extremely small. Functional limitations and social interaction also showed reciprocal prospective associations across adjacent waves.

### 4.1 Longitudinal Associations Between Diabetes and Depressive Symptoms

The prospective association between diabetes and depressive symptoms is consistent with previous cohort studies and meta‐analyses showing that diabetes is associated with an increased risk of subsequent depression [3, 33]. A previous CHARLS‐based study also reported a bidirectional association between diabetes and depressive symptoms among middle‐aged and older Chinese adults [4]. That study examined the two directions separately using time‐to‐event models [4], whereas the present analysis estimated the reciprocal paths simultaneously while accounting for prior levels of diabetes and depressive symptoms.

Importantly, the adjacent‐wave paths in our study showed interval‐specific patterns. T1 diabetes was associated with T2 depressive symptoms, whereas the corresponding T2–T3 path was not statistically significant. In the reverse direction, both adjacent‐wave paths were significant in Model 0, but only the T1–T2 path was significant in Model 4 and the logistic regression analyses. The logistic regression analyses examined incident diabetes among participants without previously reported diabetes, whereas the CLPM modelled repeated diabetes status in the full analytical sample. Differences in study populations and outcomes therefore limited direct comparisons. Evidence for the depressive symptoms‐to‐diabetes association was sensitive to model specification and follow‐up interval.

Several biological, behavioural, and psychosocial processes may underlie the bidirectional association between diabetes and depressive symptoms. In the depressive symptoms‐to‐diabetes direction, the meta‐analysis by Mezuk et al. [3] and subsequent work by Nouwen et al. [33] suggested that the hypothalamic–pituitary–adrenal axis and sympathetic nervous system, together with chronic inflammation, may contribute to insulin resistance and impaired glucose regulation. Depressive symptoms may also increase diabetes risk indirectly through physical inactivity, poor diet, smoking, poor treatment adherence, and the metabolic effects of antidepressant medications [2, 6]. In the opposite direction, the continuing demands of diabetes self‐management, including medication use, dietary regulation, glucose monitoring, and continuing engagement with healthcare services, can generate sustained psychological burden [2, 34]. Diabetes‐related complications may further reduce independence and participation in valued activities, thereby increasing vulnerability to depressive symptoms [33, 34]. These behavioural, biological, and psychosocial processes are likely to operate simultaneously and may reinforce one another, consistent with Katon's biopsychosocial model [7].

The multigroup analyses did not detect significant differences in the structural pathways across gender or age groups. Although previous studies have suggested that the association between diabetes and depressive symptoms may vary according to age and gender [3, 35], the literature remains mixed. This finding is consistent with evidence from another prospective cohort study in China, in which gender did not significantly modify the association between diabetes and incident depressive symptoms [36]. However, CHARLS did not include adults younger than 45 years, and the broad age categories used in the present study may have obscured more subtle heterogeneity.

### 4.2 Indirect Pathways Through Functional Limitations and Social Interaction

Functional limitations and social interaction statistically supported indirect pathways from T1 diabetes to T3 depressive symptoms. Diabetes is closely linked to impaired daily functioning due to complications such as peripheral neuropathy, cardiovascular disease, visual impairment, sarcopenia, and foot ulcers [11, 12]. Difficulties performing basic activities of daily living may subsequently restrict autonomy and valued activities, increase dependence on others and contribute to feelings of helplessness or loss of control, all of which may be associated with heightened vulnerability to depressive symptoms [27, 37]. Longitudinal evidence from China confirms that limitations in daily tasks are strong predictors of worsening depressive symptoms in individuals with diabetes [37]. Similarly, a U.S. study found that functional impairment mediated the association between diabetes‐related symptoms and depressive outcomes, suggesting that reduced physical capacity may amplify the psychological burden [38].

In addition, the burden of diabetes self‐management and limitations in physical functioning may also reduce opportunities or willingness to engage in supportive social activities [2, 39]. Lower social engagement may, in turn, reduce access to emotional and practical support and intensify diabetes‐related distress. Diabetes‐related distress affects approximately 36% of patients and is associated with poor self‐care, low activity, and adverse metabolic outcomes [40]. Social support is well established as a protective factor buffering these effects [41]. Conversely, negative social experiences, whether with friends, family, broader social networks, or healthcare providers, can intensify distress.

In the ML‐based CLPM, the reverse indirect pathways from depressive symptoms to diabetes through functional limitations and social interaction were statistically supported but very small. Their small magnitude indicates limited practical importance despite statistical significance, and these findings should therefore be interpreted cautiously. Other unmeasured biological and behavioural pathways may be more important in the depressive symptoms‐to‐diabetes direction, including adiposity, smoking, physical inactivity, poor diet, and reduced healthcare engagement [42, 43]. In addition, our measures of social interaction primarily captured the frequency of activity engagement and did not assess subjective loneliness, perceived support quality, or social network characteristics. Prior longitudinal evidence suggests that subjective loneliness was more strongly associated with diabetes risk than frequency of social contact [18]. The small reverse indirect effect through social interaction may partly reflect the limited scope of the measure rather than the absence of broader social mechanisms.

### 4.3 Longitudinal Associations Between Functional Limitations and Social Interaction

Functional limitations and social interaction were prospectively associated in both directions across waves. Individuals with diabetes or mobility issues often face barriers to social interaction, including greater difficulty performing daily tasks, limited access to public spaces, and illness‐related stigma, all of which may reduce willingness to engage socially [7, 44]. Conversely, reduced social interaction was associated with reduced physical activity, fewer opportunities for instrumental and emotional support, and less engagement in health‐promoting activities, potentially contributing to subsequent functional decline [45, 46]. These findings are consistent with previous evidence linking stronger social relationships with better functioning among adults with chronic conditions. For example, a longitudinal study of adults aged ≥ 65 years with chronic diseases found that improvements in the social relationship index were associated with better physical function [45]. Another cross‐sectional study in the U.S. confirmed that individuals with physical impairments were more likely to engage in fewer social, household, and outdoor activities [46].

### 4.4 Practical Implications and Limitations

The findings support integrating routine assessments of depressive symptoms, physical functioning, and social interaction into comprehensive diabetes care. Assessment of difficulties with daily activities may help identify people with diabetes who are also experiencing or may be vulnerable to psychological distress. Information about social interaction may provide additional context regarding patients' everyday resources and constraints.

Several limitations should be considered. First, the conventional CLPM does not separate stable between‐person differences from within‐person changes. Therefore, the estimated paths should be interpreted as population‐level prospective associations rather than evidence that within‐person changes in one variable produced subsequent changes in another. Second, although the three‐wave design established temporal ordering, it does not establish causal mediation because residual and time‐varying confounding could not be excluded. This qualification is especially important for reverse indirect effects, which were statistically significant but negligible in magnitude. Third, diabetes was coded 0/1 and was not declared as a categorical outcome in the ML‐based model. Coefficients involving diabetes were reported as standardised associations rather than odds ratios or risk ratios. Although the logistic sensitivity analyses explicitly modelled diabetes as a binary outcome and provided complementary evidence, they assessed only selected prospective associations and did not evaluate the robustness of the indirect effects. The very small reverse indirect effects should be interpreted cautiously. In addition, the unequal and relatively long intervals between waves may have obscured shorter‐term changes, and the CES‐D10, which assesses depressive symptoms during the preceding week, may not adequately capture persistent or recurrent symptoms over follow‐up. Differences between included and excluded participants also raise the possibility of selection bias. Diabetes, functional limitations, and social interaction were self‐reported, and the social interaction measure assessed participation frequency but not the quality or subjective experience of social relationships. Finally, residual confounding remains possible because detailed information on income, occupation, healthcare access, diabetes severity, complications, and medication use was unavailable or not included. Future studies using more frequent assessments, richer clinical and social measures, and models that distinguish within‐person change from between‐person differences, such as a random‐intercept cross‐lagged panel model (RI‐CLPM), are needed to clarify these relationships.

## 5 Conclusion

Diabetes and depressive symptoms showed reciprocal prospective associations, although support for the depressive symptoms‐to‐diabetes association was sensitive to model specification and follow‐up interval. Indirect pathways from diabetes to subsequent depressive symptoms through functional limitations and social interaction were statistically supported, whereas the reverse indirect effects were statistically significant but very small. These findings suggest that psychological, functional, and social assessments may provide complementary information in comprehensive diabetes care. Further studies with prospective intervention designs are needed to determine whether targeting these potentially modifiable factors improves health outcomes.

## Author Contributions

**Yan Tao:** writing – original draft, writing – review and editing, funding acquisition, visualisation, validation, resources, project administration, formal analysis, software, methodology, conceptualization, data curation. **Claire MacGilchrist:** writing – review and editing, visualisation, validation, conceptualization. **Ellen Kirwan:** writing – review and editing, validation, conceptualization. **Joanne E. Hurst:** writing – review and editing, visualisation. **Caroline McIntosh:** writing – review and editing, visualisation, supervision, validation, conceptualization.

## Funding

Yan Tao is supported by the China Scholarship Council (CSC) for doctoral study at the University of Galway (Grant No. 202306370011).

## Ethics Statement

The study received approval from the Institutional Review Board of Peking University (IRB00001052‐11015).

## Conflicts of Interest

The authors declare no conflicts of interest.

## Supporting information


**Figure S1:** Flowchart of participant inclusion and exclusion.
**Figure S2:** Path coefficients in stability model 1. Single‐headed arrows represented regression paths. Double‐headed arrows represented correlations. The solid line represents a significant path, and the dashed line represents a non‐significant path. All path coefficients were standardised. *p < 0.05, **p < 0.01, ***p < 0.001.
**Figure S3:** Path coefficients in the diabetes‐to‐depressive symptoms mediation model (Model 2). Single‐headed arrows represented regression paths. Double‐headed arrows represented correlations. The solid line represents a significant path, and the dashed line represents a non‐significant path. All path coefficients were standardised. *p < 0.05, **p < 0.01, ***p < 0.001.
**Figure S4:** Path coefficients in the depressive symptom‐to‐diabetes mediation model (Model 3). Single‐headed arrows represented regression paths. Double‐headed arrows represented correlations. The solid line represents a significant path, and the dashed line represents a non‐significant path. All path coefficients were standardised. *p < 0.05, **p < 0.01, ***p < 0.001.
**Figure S5:** Path coefficients from the sensitivity analysis excluding participants with baseline depressive caseness. Single‐headed arrows represented regression paths. Double‐headed arrows represented correlations. The solid line represents a significant path, and the dashed line represents a non‐significant path. All path coefficients were standardised. *p < 0.05, **p < 0.01, ***p < 0.001.
**Table S1:** Model fit indices from sensitivity analyses.
**Table S2:** Model fit indices from multigroup analysis by gender.
**Table S3:** Model fit indices from multigroup analysis by age group.

## Data Availability

The data that support the findings of this study are publicly available from the China Health and Retirement Longitudinal Study (CHARLS) upon registration and approval. Available from: https://charls.charlsdata.com/pages/data/111/zh‐cn.
