# Supplementary material for: Longitudinal Study of Diabetes and Depressive Symptoms Among Middle‐Aged and Older Adults in China: The Mediating Roles of Functional Limitations and Social Interaction
Source: Endocrinol Diabetes Metab. 2026 Nov 10;9(6):e70378. doi: 10.1002/edm2.70378 (PMC13750668; doi:10.1002/edm2.70378)
Supplement: Supplementary file 1 [file EDM2-9-e70378-s001.docx]

**Fig. S1** Flowchart of participant inclusion and exclusion

**Fig. S2** Path coefficients in stability model 1

Note: Single-headed arrows represented regression paths. Double-headed arrows represented correlations. The solid line represents a significant path, and the dashed line represents a non-significant path. All path coefficients were standardised. **p* < 0.05, ***p* < 0.01, ****p* < 0.001

**Fig. S3** Path coefficients in the diabetes-to-depressive symptoms mediation model (Model 2)

Note: Single-headed arrows represented regression paths. Double-headed arrows represented correlations. The solid line represents a significant path, and the dashed line represents a non-significant path. All path coefficients were standardised. **p* < 0.05, ***p* < 0.01, ****p* < 0.001

**Fig. S4** Path coefficients in the depressive symptom-to-diabetes mediation model (Model 3)

Note: Single-headed arrows represented regression paths. Double-headed arrows represented correlations. The solid line represents a significant path, and the dashed line represents a non-significant path. All path coefficients were standardised. **p* < 0.05, ***p* < 0.01, ****p* < 0.001

**Table S1** Model fit indices from sensitivity analyses

| Model | Model fit indices | | | | |
| --- | --- | --- | --- | --- | --- |
|  | χ2(*df*) | RMSEA | CFI | TLI | SRMR |
| Excluding participants with baseline depressive caseness | 61.632 (17) | 0.024 | 0.997 | 0.985 | 0.009 |

Note: χ2 = Chi-Square Test, df = Degrees of Freedom, RMSEA = Root Mean Square Error of Approximation, CFI = Comparative Fit Index, TLI = Tucker-Lewis Index, SRMR = Standardized Root Mean Square Residual.

**Fig. S5** Path coefficients from the sensitivity analysis excluding participants with baseline depressive caseness

Note: Single-headed arrows represented regression paths. Double-headed arrows represented correlations. The solid line represents a significant path, and the dashed line represents a non-significant path. All path coefficients were standardised. **p* < 0.05, ***p* < 0.01, ****p* < 0.001

**Table S2** Model fit indices from multigroup analysis by gender

| Model | Model fit indices | | | | |
| --- | --- | --- | --- | --- | --- |
|  | χ2(*df*) | RMSEA | CFI | TLI | SRMR |
| Unconstrained Model | 293.079 (44) | 0.042 | 0.990 | 0.961 | 0.026 |
| Constrained Model | 389.098 (82) | 0.034 | 0.987 | 0.974 | 0.029 |

Note: χ2 = Chi-Square Test, df = Degrees of Freedom, RMSEA = Root Mean Square Error of Approximation, CFI = Comparative Fit Index, TLI = Tucker-Lewis Index, SRMR = Standardized Root Mean Square Residual.

**Table S3** Model fit indices from multigroup analysis by age group

| Model | Model fit indices | | | | |
| --- | --- | --- | --- | --- | --- |
|  | χ2(*df*) | RMSEA | CFI | TLI | SRMR |
| Unconstrained Model | 198.053 (44) | 0.033 | 0.994 | 0.975 | 0.021 |
| Constrained Model | 280.760 (82) | 0.028 | 0.992 | 0.983 | 0.023 |

Note: χ2 = Chi-Square Test, df = Degrees of Freedom, RMSEA = Root Mean Square Error of Approximation, CFI = Comparative Fit Index, TLI = Tucker-Lewis Index, SRMR = Standardized Root Mean Square Residual.
